# Dynamics of proteins with different molecular structures under solution condition

**DOI:** 10.1038/s41598-020-78311-4

**Published:** 2020-12-10

**Authors:** Rintaro Inoue, Takashi Oda, Hiroshi Nakagawa, Taiki Tominaga, Tomohide Saio, Yukinobu Kawakita, Masahiro Shimizu, Aya Okuda, Ken Morishima, Nobuhiro Sato, Reiko Urade, Mamoru Sato, Masaaki Sugiyama

**Affiliations:** 1 Institute for Integrated Radiation and Nuclear Science, Kyoto University, Kumatori, Sennan-gun, Osaka, 590-0494 Japan; 2 Graduate School of Medical Life Science, Yokohama City University, Yokohama, 230-0045 Japan; 3 Department of Life Science, Rikkyo University, Nishi-Ikebukuro, Toshima-ku, Tokyo, 171-8501 Japan; 4 Hierarchical Structure Research Group, Neutron Materials Research Division, Materials Sciences Research Center, Japan Atomic Energy Agency, Tokai, Ibaraki 319-1195 Japan; 5 J-PARC Center, Japan Atomic Energy Agency, Tokai, Ibaraki 319-1195 Japan; 6 Neutron Science and Technology Center, Comprehensive Research Organization for Science and Society (CROSS), Tokai, Ibaraki 319-1106 Japan; 7 Institute of Advanced Medical Sciences, Tokushima University, Tokushima, 770-8503 Japan

**Keywords:** Biophysics, Structure determination

## Abstract

Incoherent quasielastic neutron scattering (iQENS) is a fascinating technique for investigating the internal dynamics of protein. However, low flux of neutron beam, low signal to noise ratio of QENS spectrometers and unavailability of well-established analyzing method have been obstacles for studying internal dynamics under physiological condition (in solution). The recent progress of neutron source and spectrometer provide the fine iQENS profile with high statistics and as well the progress of computational technique enable us to quantitatively reveal the internal dynamic from the obtained iQENS profile. The internal dynamics of two proteins, globular domain protein (GDP) and intrinsically disordered protein (IDP) in solution, were measured with the state-of-the art QENS spectrometer and then revealed with the newly developed analyzing method. It was clarified that the average relaxation rate of IDP was larger than that of GDP and the fraction of mobile H atoms of IDP was also much higher than that of GDP. Combined with the structural analysis and the calculation of solvent accessible surface area of amino acid residue, it was concluded that the internal dynamics were related to the highly solvent exposed amino acid residues depending upon protein’s structure.

## Introduction

All proteins are not fixed to specific configurations under solution conditions but have dynamical fluctuation; that is, they have internal dynamics^1^ which are considered relevant to the development of their functions. Hence identification of internal dynamics, such as characteristic time and spatial scales up to 1 µs and 1000 Å, are vital for revealing the mechanisms underlying protein functions^2^. Quasielastic neutron scattering (QENS) is an optimal technique for studying the dynamics of various samples in the time and spatial scales described above^2–5^. Furthermore, a notable point associated with QENS is that the incoherent neutron scattering cross-section of hydrogen (H) atoms is higher than that of other atoms in proteins. Since H atoms are embedded all over a protein, incoherent QENS (iQENS) enables the detection of internal dynamics as ensembles of H atoms. Some experimental studies of internal dynamics have applied iQENS^6^, but most samples have been in the form of powders and not solutes. Therefore, it has been highly expected that protein internal dynamics could be investigated under physiological condition (in solution). However, two key factors impeded the application of iQENS to proteins in solutions. One was the difficulty of achieving sufficient counting statistics due to the low flux of the neutron beam. The other was difficulty with decoupling internal dynamics, i.e., the translational and rotational diffusions from observed iQENS profiles. Recent upgrades of both neutron sources and state-of-the QENS spectrometers^7–10^ around the world offer better statistical data about solutions even at biologically reasonable concentrations. Namely, the first factor significantly improved and being improved at present. As for the latter factor, we would like to explain the current situation about the analyzing method in more detail. As Hong et al.^11^ pointed out, the contribution of translational diffusion and rotational diffusions, namely rigid body motion, to the observed QENS profile is dominant in solution. Such huge contribution of the rigid body motion to QENS profile hinders quantitative characterization of the remaining motion, internal dynamics. To overcome such a problematic situation, double Lorentzian function, which takes into account for the contribution of both rigid body motion and internal dynamics, was developed by Perez et al.^12^ Through the optimum selection of energy resolution and energy window of QENS spectrometer, the internal dynamics were successfully decoupled from the observed iQENS profile with this function^12–14^. In order to extend the further usability of this function, we developed a new analyzing method that provides the precise contributions of translational and rotational diffusions to the observed iQENS profile explicitly with the aid of computational technique. We then apply this newly developed method for studying the internal dynamics of two proteins, GDP and IDP.

We investigated MurD^15^ as a typical GDP, and the intrinsically disordered region (IDR) of Hef (helicase-associated endonuclease for fork-structured DNA) (Hef-IDR) as a typical IDP^16^. These proteins have similar translation and rotational diffusion constants, thus, offering an advantage for studying the effects of different molecular structures on internal dynamics. Here, we characterized and elucidated origin of internal dynamics.

## Results

### Solution structures of MurD and Hef-IDR

With the usage of recent state-of-the art software^17,18^, it is possible to reconstruct low-resolution three-dimensional structure from one-dimensional SAXS profile. Our aim for reconstruction of low-resolution three-dimensional structure from SAXS measurements is to compute the translational and rotational diffusion constants, which are used to calculate the contribution of rigid body motion to iQENS profile. We then performed SAXS measurements prior to iQENS measurements.

**Figure 1:**
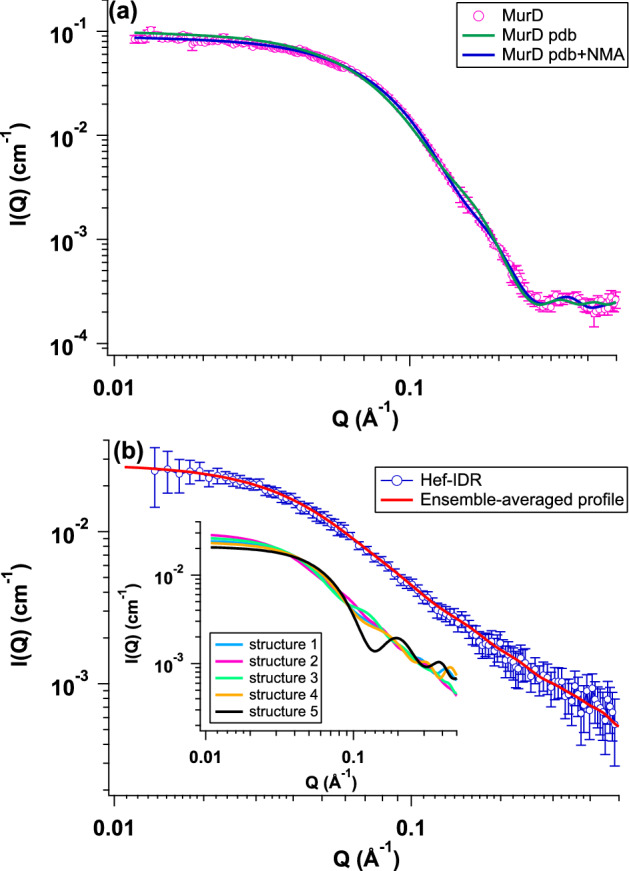
SAXS profiles of MurD and Hef-IDR. **(a)** Pink circles indicate experimental SAXS profile of MurD. Green and blue lines correspond to SAXS profiles calculated with its crystal (PDB code: 1e0d.pdb) and deformed structures by normal mode analysis (NMA), respectively. **(b)** Blue circles, experimental SAXS profile of Hef-IDR. Red line, ensemble-averaged profile of five representative structures. Inset, SAXS profiles of representative structures 1 (light blue), 2 (pink), 3 (light green), 4 (yellow) and 5 (black). This figure is prepared by the usage of IGOR Pro 6.34A (https://www.wavemetrics.com/forum/news-and-announcements/igor-634a-now-shipping).

The crystal structure of MurD (PDB code: 1e0d.pdb) deviated from that calculated from the SAXS profile (χ^2^ = 90.9)^19^. We then conducted normal mode analysis (NMA) of the crystal structure to determine the structure of MurD in solution^19^. The SAXS profile of the NMA-deformed structure reproduced the experimental SAXS profile of MurD (χ^2^ = 5.4) as shown in, Fig. 1a. Contrary to GDP, IDP has largely different configurations^20^. Following this notion, we searched the configurations of Hef-IDR with MultiFoXS^18^, which enables structural modeling and generated the representative structures 1–5. Considering that the populations of structures 1–5 were 0.116, 0.442 0.267, 0.053, and 0.122, respectively, the ensemble-averaged profile reproduced the experimental profile (χ^2^ = 1.9), as shown in Fig. 1b.

### Internal dynamics of MurD and Hef-IDR

**Figure 2:**
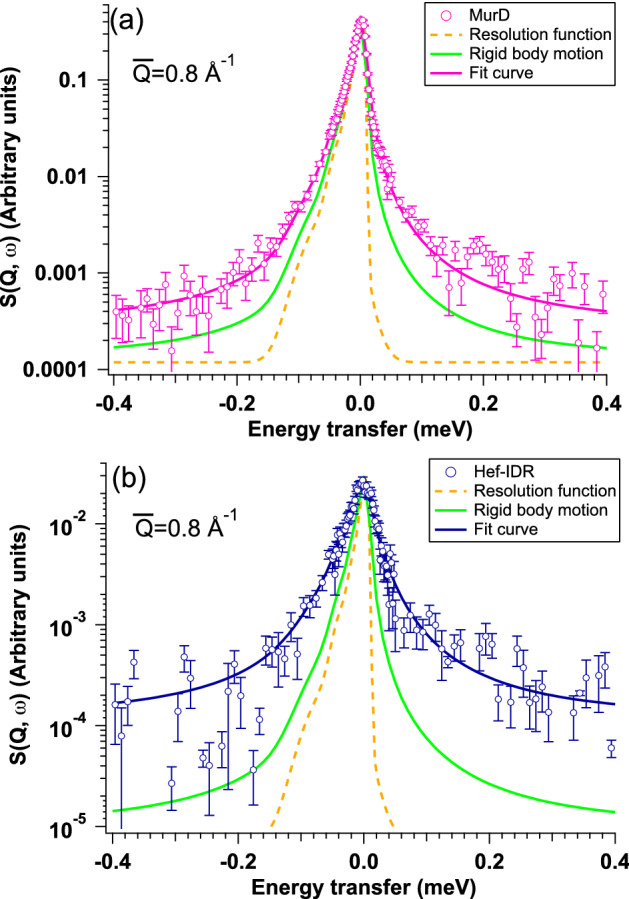
*S*(*Q*, ω)s of MurD and Hef-IDR. **(a)** Pink circles show experimental *S*(*Q*, ω) of MurD at = $\bar{Q}$0.80 Å^−1^. Broken orange, solid green and solid pink lines correspond to the resolution function, *S*(*Q*, ω) originating from rigid body motion and curve fit with Eq. (5), respectively. **(b)** Blue circles show experimental *S*(*Q*, ω) of Hef-IDR at = $\bar{Q}$0.80 Å^−1^. Broken orange, solid green, and solid blue lines correspond to the resolution function, *S*(*Q*, ω) originating from rigid body motion and curve fit with Eq. (5), respectively. This figure is prepared by the usage of IGOR Pro 6.34A (https://www.wavemetrics.com/forum/news-and-announcements/igor-634a-now-shipping).

We conducted iQENS measurements using a BL02 DNA inverted geometry time-of-flight spectrometer^21^ installed at the Materials and Life Science Experimental Facility in J-PARC, Tokai, Japan. Since the detailed solution conditions are helpful for both the analysis and interpretation of iQENS profiles, we then describe them prior to explaining the iQENS results. Hef-IDR solution was prepared in a D_2_O buffer comprising 10 mM HEPES pD 7.1, 100 mM NaCl and 0.1 mM EDTA and the solution of Hef-IDR at the concentration of 8.0 mg/mL was used for iQNES measurement. MurD solution was prepared in a D_2_O buffer containing 20 mM Tris-DCl pD 7.2, 200 mM NaCl, 10 mM dithiothreitol and 0.05% NaN_3_ and the solution of MurD at the concentration of 52.0 mg/mL was utilized for iQNES measurement. The observed two-dimensional dynamic scattering laws (*S*(*Q*, *ω*)) (Fig. S1), where *ω* corresponds to the energy transfer frequency of a neutron through *ħω* (*ħ*: Dirac’s constant), were divided into six *Q* regions as follows [(1, ($\stackrel{-}{Q}$ = 0.21 Å^−1^, 0.13 Å^−1^ ≤ *Q* ≤ 0.30 Å^−1^); 2, ($\stackrel{-}{Q}$ = 0.40 Å^−1^, 0.30 Å^−1^ ≤ *Q* ≤ 0.50 Å^−1^), 3, ($\stackrel{-}{Q}$ = 0.60 Å^−1^, 0.50 Å^−1^ ≤ *Q* ≤ 0.70 Å^−1^); 4, ($\stackrel{-}{Q}$ = 0.80 Å^−1^, 0.70 Å^−1^ ≤ *Q* ≤ 0.90 Å^−1^); 5, ($\stackrel{-}{Q}$ = 1.0 Å^−1^, 0.90 Å^−1^ ≤ *Q* ≤ 1.1 Å^−1^); and 6, ($\stackrel{-}{Q}$ = 1.2 Å^−1^, 1.1 Å^−1^ ≤ *Q* ≤ 1.3 Å^−1^)). By averaging the two-dimensional *S*(*Q*, *ω*) over each region, one dimensional *S*(*Q*, *ω*) at six values were gained for MurD and Hef-IDR (Fig. S2). We then analyzed their *S*(*Q*, *ω*) and Fig. 2 shows *S*(*Q*, *ω*) at = 0.80 Å^−1^, as a representative. Compared with the resolution function (orange broken lines), the spectra broadened in both samples, indicating that the motion of the proteins was anharmonic.

Since the observed *S*(*Q*,* ω*) consists of three dynamics, translational diffusion, rotational diffusion and internal dynamics^22^, we had to decompose the observed *S*(*Q*, *ω*) into them. The sum of translational and rotational diffusions^12^ named as a rigid body motion and then its contribution to *S*(*Q*, *ω*), *S*(*Q*, *ω*)_rb_ is given by following functions:

$${S\left(Q,\omega \right)}_{\mathrm{rb}}={S\left(Q,\omega \right)}_{\mathrm{trans}}\otimes {S\left(Q,\omega \right)}_{\mathrm{rot}}$$

$${S\left(Q,\omega \right)}_{\mathrm{trans}}=\frac{1}{\pi }\frac{{D}_{t}{{H}_{t}Q}^{2}}{{\omega }^{2}+{\left({D}_{t}{{H}_{t}Q}^{2}\right)}^{2}}$$

$${S\left(Q,\omega \right)}_{\mathrm{rot}}=\sum_{l=0}^{\infty }{\int }_{0}^{{R}_{h}}\left(2l+1\right){j}_{l}^{2}\left(Qr\right)\cdot 4\pi {r}^{2}dr\cdot \frac{1}{\pi }\cdot \frac{l\left(l+1\right){D}_{r}}{{\omega }^{2}+{\left(l\left(l+1\right){D}_{r}\right)}^{2}}$$

$${S\left(Q,\omega \right)}_{\mathrm{rb}}={S\left(Q,\omega \right)}_{\mathrm{trans}}\otimes {S\left(Q,\omega \right)}_{\mathrm{rot}}=\sum_{l=0}^{\infty }{\int }_{0}^{{R}_{h}}\left(2l+1\right){j}_{l}^{2}\left(Qr\right)\cdot 4\pi {r}^{2}dr\cdot \frac{1}{\pi }\cdot \frac{\left(l\left(l+1\right){D}_{r}+{D}_{t}{{H}_{t}Q}^{2}\right)}{{\omega }^{2}+{\left(l\left(l+1\right){D}_{r}+{D}_{t}{H}_{t}{Q}^{2}\right)}^{2}}$$

1 $${S\left(Q,\omega \right)}_{\mathrm{rb},\mathrm{ex}}={S\left(Q,\omega \right)}_{\mathrm{rb}}\otimes \mathrm{Res}\left(Q,\omega \right) ,$$

${S\left(Q,\omega \right)}_{\mathrm{rb}}\, \mathrm{is\, the} \,S\left(Q,\omega \right) \mathrm{of\, rigid\, body\, motion},$ ${S\left(Q,\omega \right)}_{\mathrm{trans}}\,\mathrm{ is\, the} \,S\left(Q,\omega \right) \mathrm{of\, translational\, diffusion},$ ${S\left(Q,\omega \right)}_{\mathrm{rot}}\,\mathrm{ is\, the }\, S\left(Q,\omega \right) \mathrm{of\, rotational\, diffusion},$ $\mathrm{Res}\left(Q,\omega \right)\, \mathrm{is\, the \,resolution\, function},$ ${S\left(Q,\omega \right)}_{\mathrm{rb},\mathrm{ex}}\, \mathrm{is\, the}\, {S\left(Q,\omega \right)}_{\mathrm{rb}} \mathrm{convoluted\, with\, a\, resolution\, function},$*D*_*t*_ is the translational diffusion constant, *H*_*t*_ is the hydordynamic function to translational diffusion constant, *R*_*h*_ is the hydrodynamic radius, r is the the distance from the center of hard sphere where an isotropic diffusion was assumed, *j*_*l*_ is the lth order sperical Bessel function, $\otimes$ is the convolution operator.

The *D*_t_ and *D*_r_ values of MurD and Hef-IDR were computed using “HYDROPRO”^23^. The *D*_t_ and *D*_r_ values of MurD determined using the single structure resolved by the SAXS measurements were 6.42 × 10^–7^ cm^2^/s and 5.34 × 10^6^ s^−1^, respectively. As described above, the SAXS profile of Hef-IDR reproduced the ensemble-averaged profile over five structures. For Hef-IDR, the *D*_t_ values of structures 1–5 of Hef-IDR were calculated to 6.98 × 10^–7^ cm^2^/s, 6.47 × 10^–7^ cm^2^/s, 6.68 × 10^–7^ cm^2^/s, 7.05 × 10^–7^ cm^2^/s and 7.23 × 10^–7^ cm^2^/s, respectively. The *D*_r_ values of structures 1–5 of Hef-IDR were calculated to 5.73 × 10^6^ s^−1^, 5.42 × 10^6^ s^−1^, 5.03 × 10^6^ s^−1^, 5.85 × 10^6^ s^−1^ and 6.54 × 10^6^ s^−1^, respectively. Five sets of separately calculated *D*_t_ and *D*_r_ values were averaged depending on their populations in the ensemble-averaged profile, and the averaged *D*_t_ and *D*_r_ values were 6.71 × 10^–7^ cm^2^/s and 5.51 × 10^6^ s^−1^, respectively, for Hef-IDR. It was confirmed that the diffusion constants of MurD and Hef-IDR were almost the same. Considering the concentration of MurD and Hef-IDR used for iQENS measurements, we also calculated *H*_t_ for both samples. *H*_t_ values were found to be 0.71 and 0.99 for MurD and Hef-IDR, respectively. The detail of calculation of *H*_t_ values should be referred to Supplementary Materials. Substituting the *D*_t_ and *D*_r_ values into Eq. (1), *S*(*Q*, *ω*)_rb, ex_s were calculated for MurD and Hef-IDR, respectively. *S*(*Q*, *ω*)_rb, ex_s $\stackrel{-}{Q}$ at = 0.80 Å^−1^ are shown as green lines in Fig. 2. Because *S*(*Q*, *ω*)s of both samples could not be described only as rigid body motions, the detection of internal dynamics was implied.

A modified function was then considered to reproduce the observed *S*(*Q*, *ω*). Sarter et al*.*^14^ reported that observed *S*(*Q*, *ω*) could be expressed as a convolution of two dynamic scattering functions *S*(*Q*, *ω*)_rb_ and *S*(*Q*, *ω*)_int_, which describes the internal dynamics given by Eq. (2):

2 $${S\left(Q,\omega \right)}_{\mathrm{mod}}=\left[\left(1-A\left(Q\right)\right){S\left(Q,\omega \right)}_{\mathrm{int}}+A\left(Q\right)\delta \left(\omega \right)\right]\otimes {S\left(Q,\omega \right)}_{\mathrm{rb}} ,$$

where *δ*(*ω*) and *A*(*Q*) correspond to the delta function and elastic incoherent structure factor, respectively. For the simplification of calculation, it is assumed that the *S*(*Q*, *ω*)_int_ is described by a single Lorentz function as follows.

3 $${S\left(Q,\omega \right)}_{\mathrm{int}}=\frac{1}{\pi }\frac{\Gamma }{{\Gamma }^{2}+{\upomega }^{2}} ,$$

where *Γ* indicates the relaxation rate of internal dynamics. By substituting *S*(*Q*, *ω*)_rb_ into Eqs. (2) and  (4) is obtained as follows:

4 $${S\left(Q,\omega \right)}_{\mathrm{mod}}=\left[\left(1-A\left(Q\right)\right)\sum_{l=0}^{\infty }{\int }_{0}^{{R}_{h}}\left(2l+1\right){j}_{l}^{2}\left(Qr\right)\cdot 4\pi {r}^{2}dr\cdot \frac{1}{\pi }\cdot \frac{\left(l\left(l+1\right){D}_{r}+{D}_{t}{H}_{t}{Q}^{2}+\Gamma \right)}{{\omega }^{2}+{\left(l\left(l+1\right){D}_{r}+{D}_{t}{H}_{t}{Q}^{2}+\Gamma \right)}^{2}}+\,A\left(Q\right)\sum_{l=0}^{\infty }{\int }_{0}^{{R}_{h}}\left(2l+1\right){j}_{l}^{2}\left(Qr\right)\cdot 4\pi {r}^{2}dr\cdot \frac{1}{\pi }\cdot \frac{\left(l\left(l+1\right){D}_{r}+{D}_{t}{{H}_{t}Q}^{2}\right)}{{\omega }^{2}+{\left(l\left(l+1\right){D}_{r}+{D}_{t}{H}_{t}{Q}^{2}\right)}^{2}}\right] .$$

Taking into consideration of fast dynamics such as the rotation of methyl groups^24^ in the modified fit function, we also introduced the contribution of a flat background (*B*(*Q*)). Finally, the following modified fit function was obtained:

5 $${S\left(Q,\omega \right)}_{\mathrm{mod},\mathrm{ex}}={S\left(Q,\omega \right)}_{\mathrm{mod}}\otimes R\left(Q,\omega \right)+B\left(Q\right)$$

where *S*(*Q*, *ω*)_mod,ex_ corresponds to *S*(*Q*, *ω*)_mod_ convoluted with a resolution function. The pink and blue lines in Fig. 2 show the results of fits with Eq. (5) for MurD and Hef-IDR, respectively, and both *S*(*Q*, *ω*)s were appropriately described by this modified function.

**Figure 3:**
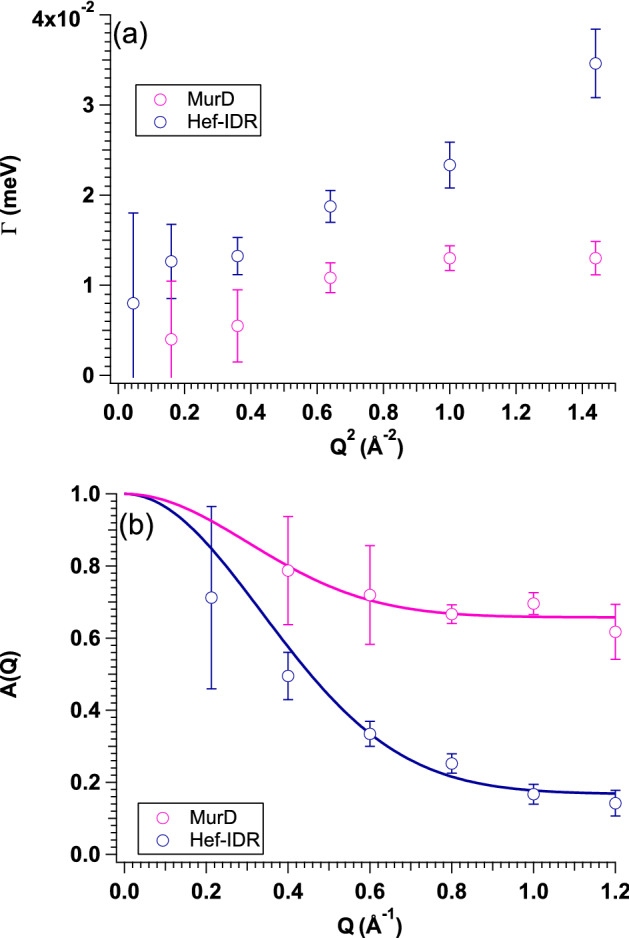
*Q*^2^ dependences of *Γ* values and *A*(*Q*)s. (a) *Q*^2^ dependences of *Γ* values of MurD (pink circle) and Hef-IDR (blue circle), respectively. (b) *Q*^2^ dependences of *A*(*Q*)s of MurD (pink circle) and Hef-IDR (blue circle), respectively. Solid lines correspond to the fits with Eq. (6) for MurD (pink line) and Hef-IDR (blue line), respectively. This figure is prepared by the usage of IGOR Pro 6.34A (https://www.wavemetrics.com/forum/news-and-announcements/igor-634a-now-shipping).

Figure 3a shows the *Q*^2^ dependence of *Γ* values from both samples. The *Γ* values were larger for Hef-IDR than MurD, meaning that the averaged internal dynamics were faster for Hef-IDR than MurD. Namely, we succeeded to exhibit the difference of internal dynamics between GDP and IDP quantitatively through the application of newly developed analyzing method to the observed *S*(*Q, ω*) profiles. Prior to the further detailed analysis of internal dynamics, we briefly explain the observable H atoms of proteins in iQENS measurements. Both MurD and Hef-IDR solution samples were prepared in D_2_O buffer to reduce the scattering signal from solvent. Under such sample condition, the exchangeable H atoms in both samples have already been exchanged with D atoms from the solvent. Therefore, the H atoms detected by iQENS are comprised of non-exchangeable H atoms in both samples. To consider the origin of the difference in the internal dynamics between GDP and IDP, we focused on the mobility of H atoms embedded on the peptide chains. We then analyzed the *Q* dependence of the elastic incoherent structure factor (*A*(*Q*)) (Fig. 3b) because the mean square displacement (< *u*^2^ >) of mobile H atoms within a protein can be determined using the following equation^14,25^:

6 $$A\left( Q \right) \, = p*{\text{exp}}\left( { - < u^{{2}} > Q^{{2}} } \right) + \left( {{1} - p} \right),$$

where the value of *p* corresponds to the fraction of mobile H atoms. The calculated values for < *u*^2^ > and *p* were 2.1 ± 0.4 Å^2^ and 0.33 ± 0.07, respectively, for MurD, and 2.1 ± 0.2 Å^2^ and 0.85 ± 0.05 respectively, for Hef-IDR. Although < *u*^2^ > was not affected by the different molecular structures, the fraction of mobile H atoms was higher in Hef-IDR than that in MurD. It should be an origin of difference of internal dynamics between them.

## Discussion

We considered the difference in *p* values between MurD and Hef-IDR. It is considered that the H atoms with high mobility are considered to be located at the surface of protein based on theoretical shell model^26^. In consistent with this idea, Zanotti et al.^27^ also reported that peripheral water-protein interaction affected the internal dynamics of protein through the combination of QNES and ^13^C-NMR. To clarify H atoms in MurD and Hef-IDR that were exposed to solvent, we obtained their solution scattering data using SAXS. The mean solvent accessible surface areas of the amino acid residues of MurD and Hef-IDR with their solution structures determined by GETAREA^28^ (probe particle radius of 1.4 Å), were 44.1 and 117.2 Å^2^, respectively. It implies that the mean value of SASA of Hef-IDR was higher than that of MurD. From the normal mode analysis for MurD, it was revealed that higher SASA possessed higher mobility from NMA (refer to Fig. S3). It is considered that there exist the relationship between internal dynamics and SASA. Then, we adopted the idea that amino acid residues exposed to a solvent could affect the internal dynamics. In the following, we explain our idea in more detail.

**Figure 4:**
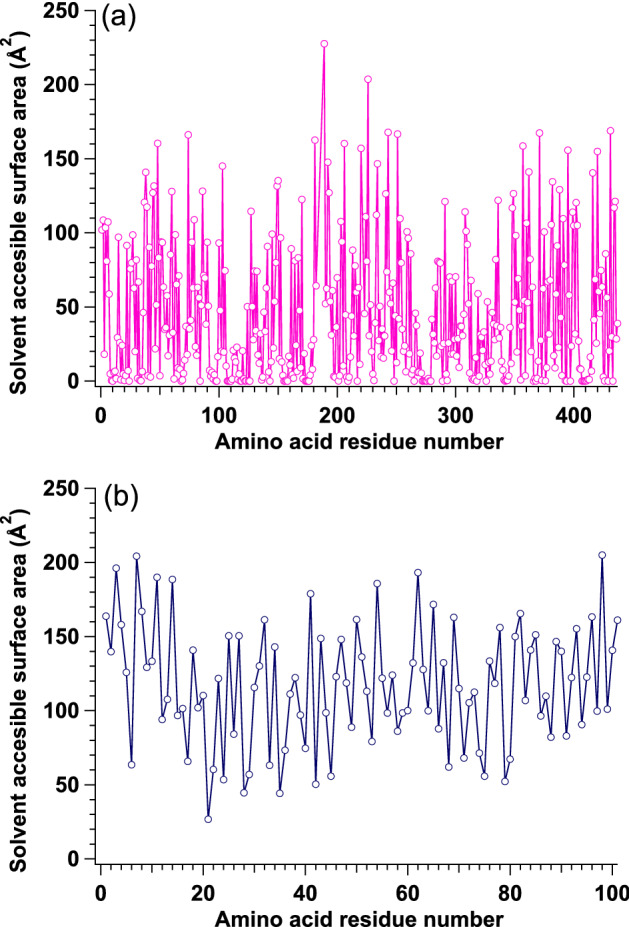
Solvent accessible surface area as function of amino acid residue numbers. Solvent accessible surface area as function of numbers of amino acid residues in **(a)** MurD and **(b)** Hef-IDR. This figure is prepared by the usage of IGOR Pro 6.34A (https://www.wavemetrics.com/forum/news-and-announcements/igor-634a-now-shipping).

**Figure 5:**
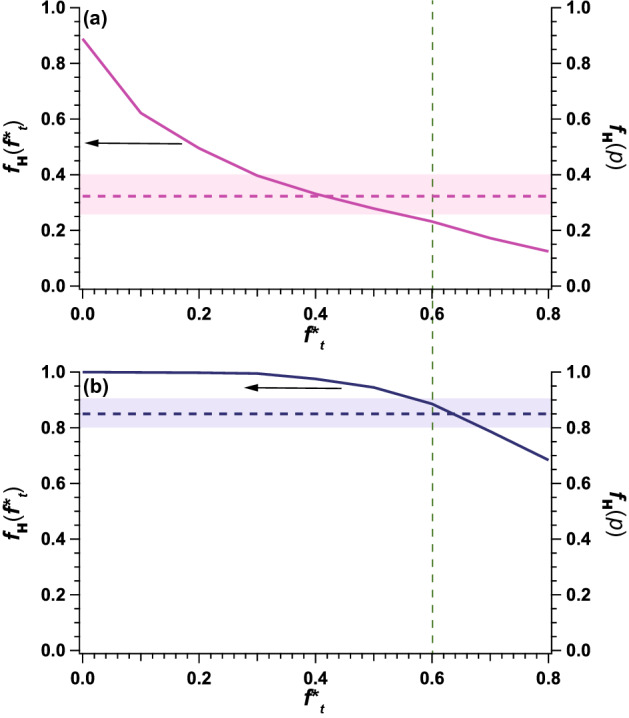
Fraction of surface H_nex_ exposed to solvent (*r*_H_) as a function of factor for surface area of amino acid residues (= f*_t_). Green dashed line, f*_t_ = 0.6. **(a)** *r*_H_ of MurD as a function of f*_t_ (purple dotted line) (left axis). Pink dotted line, *p* value *r*_H_(*p*) of MurD (right axis). Pink shaded zone shows error of *r*_H_(*p*) of MurD. **(b)** *r*_H_ of Hef-IDR as a function of f*_t_ (blue dotted line) (left axis). Blue dotted line, *r*_H_(*p*) of Hef-IDR (right axis). Blue shaded zone shows error of *r*_H_(*p*) of Hef-IDR. This figure is prepared by the usage of IGOR Pro 6.34A (https://www.wavemetrics.com/forum/news-and-announcements/igor-634a-now-shipping) and Adobe Illustrator CC 2015.2.1 (19.2.1) (https://www.adobe.com/jp/products/illustrator.html).

1. Under the assumption that a shape of amino acid residue is sphere, the entire surface area (*S*_whole_) of each amino acid residue was calculated from its volume.
2. The number of *H*_nex_ in the entire protein, *N*_whole_, was calculated^29^.
3. The solvent accessible surface area (*S*_solvent_) of each amino residue was calculated for both MurD and Hef-IDR as shown in Fig. 4.
4. The fractions of solvent exposed surface area to the entire surface area (*S*_solvent_/*S*_whole_) were defined as *f*. and *f* values were calculated for all the constituting amino acid residues for both MurD and Hef-IDR.
5. To judge whether a given amino acid residue is located at the solvent or not, we set the threshold *f* value (f*_t_) as the quantitative criteria: Here, the amino acid residue with *f* values exceeding f*_t_ (*f* > f*_t_) is regarded to be exposed to the solvent and named as *a*_exp_.
6. For each setting f*_t_ value (0.0 ~ 0.8), the entire amino acid resides were classified and then the number of non-exchangeable atoms (H_nex_) in the *a*_exp_ (*N*_surface_(f*_t_ )) was calculated.
7. The number ratio *r*_H_(f*_t_ ) (= *N*_surface_(f*_t_ )/*N*_whole_) was calculated. Pink and blue lines in Fig. 5 indicate the results for MurD and Hef-IDR, respectively.
8. Because exchangeable H atoms of protein in D_2_O were replaced with deuterium atoms, the iQENS of the protein was dominated by mobile non-exchangeable H atoms that are mainly located in amino acid residues exposed to solvent. This means that the *p* values should agree with the *r*_H_ value. In this procedure, we should find the optimum f*_t_ value that reproduce the *r*_H_ (*p*) values from both MurD and Hef-IDR simultaneously. For this purpose, we firstly calculated the following χ^2^ values against *r*_H_ (*p*) for MurD (χ^2^_m_(f*_t_ )) and Hef-IDR (χ^2^_h_(f*_t_ )), respectively.
  $${\chi }_{m}^{2}\left({f}_{t}^{*}\right)={\left(\frac{{r}_{H,m}\left({f}_{t}^{*}\right)-{r}_{H,m}\left(p\right)}{{\sigma }_{H,m}\left(p\right)}\right)}^{2}$$
  7 $${\chi }_{h}^{2}\left({f}_{t}^{*}\right)={\left(\frac{{r}_{H,h}\left({f}_{t}^{*}\right)-{r}_{H,h}\left(p\right)}{{\sigma }_{H,h}\left(p\right)}\right)}^{2}$$

where *r*_H,m_(f*_t_), *r*_H,m_(*p*), σ_H,m_(*p*), *r*_H,h_(f*_t_ ), *r*_H,h_(*p*), σ_H,h_(*p*) correspond to *r*_H_(f*_t_) from MurD, *r*_H_ (*p*) value from MurD, error value of *r*_H_ (*p*) value from MurD, *r*_H_(f*_t_) from Hef-IDR, *r*_H_ (*p*) value from Hef-IDR, and error value of *r*_H_ (*p*) value from Hef-IDR, respectively. As a next step, we named the sum of χ^2^_m_(f*_t_) and χ^2^_h_(f*_t_) as a total χ^2^ (χ^2^_tot_(f*_t_)). It is considered that optimum f*_t_ value could be determined by finding the condition where χ^2^_tot_(f*_t_) exhibited the lowest value. We then plotted χ^2^_tot_(f*_t_) in Figs. 1–3 and χ^2^_tot_(f*_t_) exhibited the smallest value at f*_t_ = 0.6. We then concluded that f*_t_ value of 0.6 satisfied the value calculated with SAXS and that observed using iQENS.

All steps are schematically summarized in Fig. S5.

**Figure 6:**
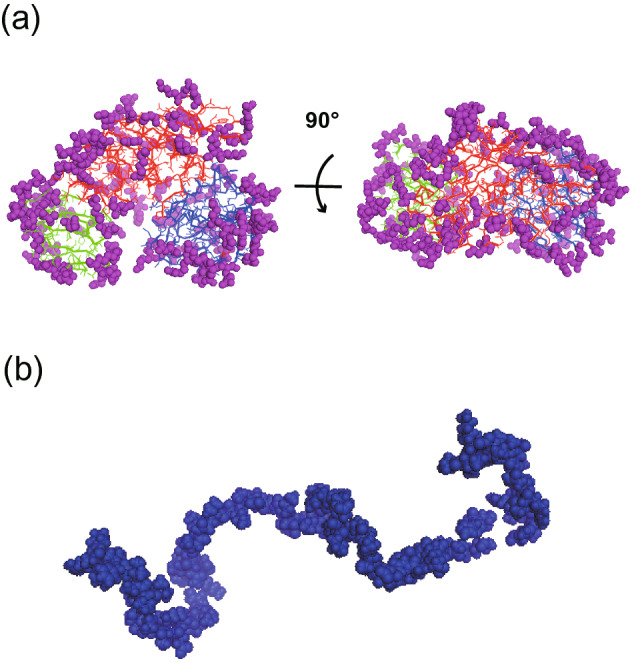
Schematic view of solvent-exposed amino acid residues for MurD and Hef-IDR when f*_t_ = 0.6. **(a)** In the case of f*_t_ = 0.6, the amino acid residues, which are located in solvent accessible surface area, were depicted for MurD by purple spheres. The domain 1, 2, 3 were depicted by green, red and blue sticks, respectively. **(b) **In the case of f*_t_ = 0.6, the amino acid residues, which are located in solvent accessible surface area, were depicted for Hef-IDR by blue spheres. This figure is prepared by the usage of Adobe Illustrator CC 2015.2.1 (19.2.1) (https://www.adobe.com/jp/products/illustrator.html) and PyMOL 1.8.4.0 (https://pymol.org/2/).

 Figure 6 shows a schema of amino acid residues in both MurD and Hef-IDR located in surface area that can access the solvent under conditions of f*_t_ = 0.6. Such amino acid residues were notably segregated only on the surface of MurD, but these were distributed and chained within the entire structure of Hef-IDR. These findings indicated that the amino acid residues at a surface with access to a solvent is responsible for the internal dynamics of proteins depending on their molecular structures. Thanks to the application of the newly developed analyzing method, we could discuss the difference of internal dynamics of GDP and IDP quantitatively. Furthermore, we could reach the present conclusion that can interpret the internal dynamics of GDP and IDP without inconsistency.

## Summary

The internal dynamics of two proteins, globular domain protein (GDP) and intrinsically disordered protein (IDP) in solution, were studied by measuring incoherent quasielastic neutron scattering (iQENS) with state-of-the art spectrometer QENS spectrometer and analyzing them with a newly developed method assisted by computational technique. It was clarified that the average relaxation rate of internal dynamics in IDP was larger than that of GDP quantitatively. From the further detailed analyzes, the fraction of mobile hydrogen (H) atoms of IDP was higher than that of GDP. Calculation of the solvent accessible surface areas per amino acid residues revealed that the fraction of highly solvent exposed H atoms was related to the fraction of mobile H atoms. Then, present iQENS studies clarified that non-exchangeable H atoms that are mainly located in amino acid residues exposed to solvent was relevant to the internal dynamics depending upon protein’s structures. It is strongly expected that our newly developed analyzing method should offer further promising opportunity for characterizing the functionally relevant internal dynamics of protein in solution.

## Materials and methods

### Sample preparation of Hef-IDR

*Escherichia coli* cells were grown at 37 °C in Luria–Bertani (LB) culture medium containing 50 µg/mL of ampicillin and 34 µg/mL of chloramphenicol^16^. When the culture reached an optical density of 0.4–0.6 at 660 nm, Hef-IDR expression was induced by adding isopropyl β-d-thiogalactopyranoside (IPTG) at a final concentration of 1 mM for 16 h at 25 °C. The cells were disrupted by sonication on ice. After centrifugation for 30 min at 20,400 × *g*, the supernatant was heated at 80 °C for 20 min and the heat resistant fraction was harvested. The supernatant was fractionated by cation exchange chromatography using a HiTrap SP column (GE healthcare science), and a linear gradient of 0–1 M NaCl. The protein fractions were loaded onto a HiLoad 26/600 Superdex 200 pg column with 10 mM HEPES, pH 7.5, 350 mM NaCl. To inactivate contaminating proteases, the eluate containing protein was heated at 80 °C for 10 min, then flash cooled in liquid nitrogen. Thereafter, Hef-IDR was placed in a D_2_O buffer comprising 10 mM HEPES pD 7.1, 100 mM NaCl and 0.1 mM EDTA and dialyzed against a buffer solution before SAXS and iQENS measurements. Hef-IDR solution samples were prepared in D_2_O buffer to reduce the scattering signal from solvent. Under such sample condition, the exchangeable H atoms in Hef-IDR have already been exchanged with D atoms from the solvent.

### Sample preparation of MurD

*Escherichia coli* MurD was cloned into pGBHPS, expressed in *E. coli* BL21 (DE3), and purified as described^30^. Cells were grown at 37 °C until they reached an optical density of 0.7–0.8 at 600 nm. Protein expression was induced by incubating the cells for 12 h at 25 °C in a final IPTG concentration of 0.5 mM, then the cells were sonicated on ice. Proteins were purified from the sonicate using His-Tag purification columns (GE Healthcare), followed by tag removal with HRV3C protease. Thereafter, MurD was placed in a D_2_O buffer containing 20 mM Tris-DCl pD 7.2, 200 mM NaCl, 10 mM dithiothreitol and 0.05% NaN_3_ and dialyzed against a buffer solution before SAXS and iQENS measurements. MurD solution samples were prepared in D_2_O buffer to reduce the scattering signal from solvent. Under such sample condition, the exchangeable H atoms in MurD have already been exchanged with D atoms from the solvent.

### Small-angle X-ray scattering (SAXS) measurements

SAXS measurements of Hef-IDR (3.4 mg/mL) were performed with a BioSAXS 1000 system mounted on a MicroMax007HF X-ray generator (Rigaku, Tokyo, Japan) at 25 °C and a PILATUS100K detector (DECTRIS, Baden-Dättwil, Switzerland) located 485 mm from the sample. The X-ray wavelength was 1.542 Å. One-dimensional scattering data (*I*(*Q*)) were obtained by radial averaging. Scattered intensity was converted into absolute scatter intensity, and calibrated based on the scatter intensity of water (*I*(*Q*)_water_ = 1.632 × 10^–2^ cm^−1^). Data were processed using SAXSLab (Rigaku) and the ATSAS package^17,31^.

SAXS measurements of MurD (3.0 mg/mL) at 25 °C were performed with a NANOPIX (Rigaku, Tokyo, Japan). X-rays emanating from a high-brilliance point-focused X-ray generator (MicroMAX-007HF, Rigaku, Tokyo, Japan) were focused using a confocal mirror (OptiSAXS) and collimated with a confocal multilayer mirror and a two-pinhole collimation system with lower parasitic scattering. The scattered X-rays were detected using a two-dimensional (2D) HyPix-6000 semiconductor detector (Rigaku, Tokyo, Japan). We covered the *Q* range (0.015–0.5 Å^−1^) by measuring SAXS profiles at sample-to-detector distances (SDD) of 1320 and 300 mm. One-dimensional *I*(*Q*) values were obtained by radial averaging the 2D scattering patterns. The scatter intensity from the protein was converted to an absolute scale by comparison with the scatter intensity of water. All data were reduced and processed using SAngler^32^.

### iQENS measurement

We measured iQENS measurements using an inverted geometry time-of-flight spectrometer (BL02 DNA) installed^21^ at the Materials and Life Science Experimental Facility (MLF) in J-PARC, Tokai, Japan. The magnitude of the scattering vector *Q* (*Q* = 4πsinθ/λ_f_, where 2θ and λ_f_ = 6.26 Å are the scattering angle and the wavelength of the analyzed neutron, respectively) ranged from 0.12 to 1.78 Å^−1^. Samples were loaded into double-cylindrical aluminum cells (outer diameter: 14 mm, inner diameter: 13 mm, height: 45 mm) under a helium atmosphere. The resolution function was determined from the measurement of vanadium at 298 K and the calculated energy resolution (*δE*) was 12 µeV. Solutions of Hef-IDR and MurD (8.0 and 52.0 mg/mL, respectively) were measured at 25 °C. Dynamic scattering laws from D_2_O buffer were subtracted from those of protein solutions based on their volume fractions to obtain the protein dynamics.

## Supplementary Information

Supplementary Figures.

## Data Availability

The datasets generated and analyzed during the current study are available from the corresponding authors on reasonable request.
