# Supplementary material for: Dynamics of proteins with different molecular structures under solution condition
Source: Sci Rep. 2020 Dec 10;10:21678. doi: 10.1038/s41598-020-78311-4 (PMC7728768; doi:10.1038/s41598-020-78311-4)
Supplement: Supplementary file 1 — Supplementary Figures. [file 41598_2020_78311_MOESM1_ESM.pdf]

Supplementary Materials

Dynamics of proteins with different molecular structures under solution condition

Rintaro Inoue[1†*], Takashi Oda[2,3†], Hiroshi Nakagawa[4.5†], Taiki Tominaga[6], Tomohide Saio[7], Yukinobu Kawakita[5], Masahiro Shimizu[1], Aya Okuda[1], Ken Morishima[1], Nobuhiro Sato[1], Reiko Urade[1], Mamoru Sato[2*] and Masaaki Sugiyama[1*]

[1]*Institute for Integrated Radiation and Nuclear Science, Kyoto University, Kumatori, Sennan-gun, Osaka 590-0494 JAPAN.*

[2]*International Graduate School of Arts and Sciences, Yokohama City University, Yokohama 230-0045, JAPAN.*

[3]*Department of Life Science, Rikkyo University, Nishi-Ikebukuro, Toshima-ku, Tokyo 171–8501, JAPAN.*

[4]*Hierarchical Structure Research Group, Neutron Materials Research Division, Materials Science Research Center, Japan Atomic Energy Agency, Tokai, Ibaraki 319-1195, JAPAN.*

[5]*Materials & Life Science Division, J-PARC Center, Tokai, Ibaraki 319-1195, JAPAN.*

[6]*Neutron Science and Technology Center, Comprehensive Research Organization for Science and Society (CROSS), Tokai, Ibaraki 319-1106, JAPAN.*

[7]*Department of Chemistry, Faculty of Science, Hokkaido University, Sapporo, 060-0810, JAPAN.*

[†]Equal contribution

*Corresponding authors. R. I. rintaro@rri.kyoto-u.ac.jp, M. Sato msato@yokohama-cu.ac.jp,

M. Sugiayama sugiyama@rri.kyoto-u.ac.jp

This PDF file includes:

Fig. S1-S5 and the calculation of H_t and H_r

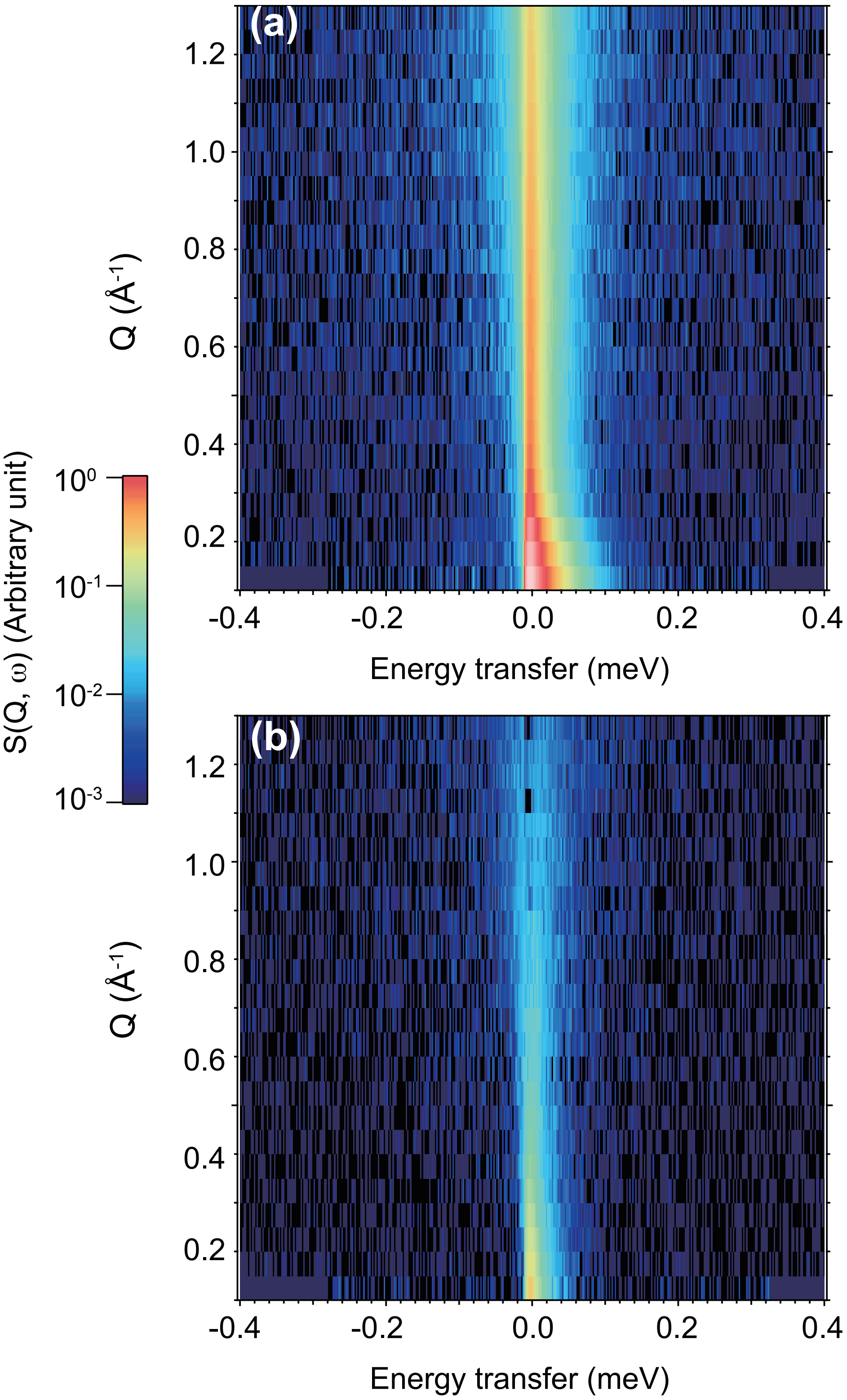

Fig. S1. Two dimensional $S(Q, \omega)$s from two samples.

(a) Two dimensional $S(Q, \omega)$ of MurD. (b) Two dimensional $S(Q, \omega)$ of Hef-IDR.

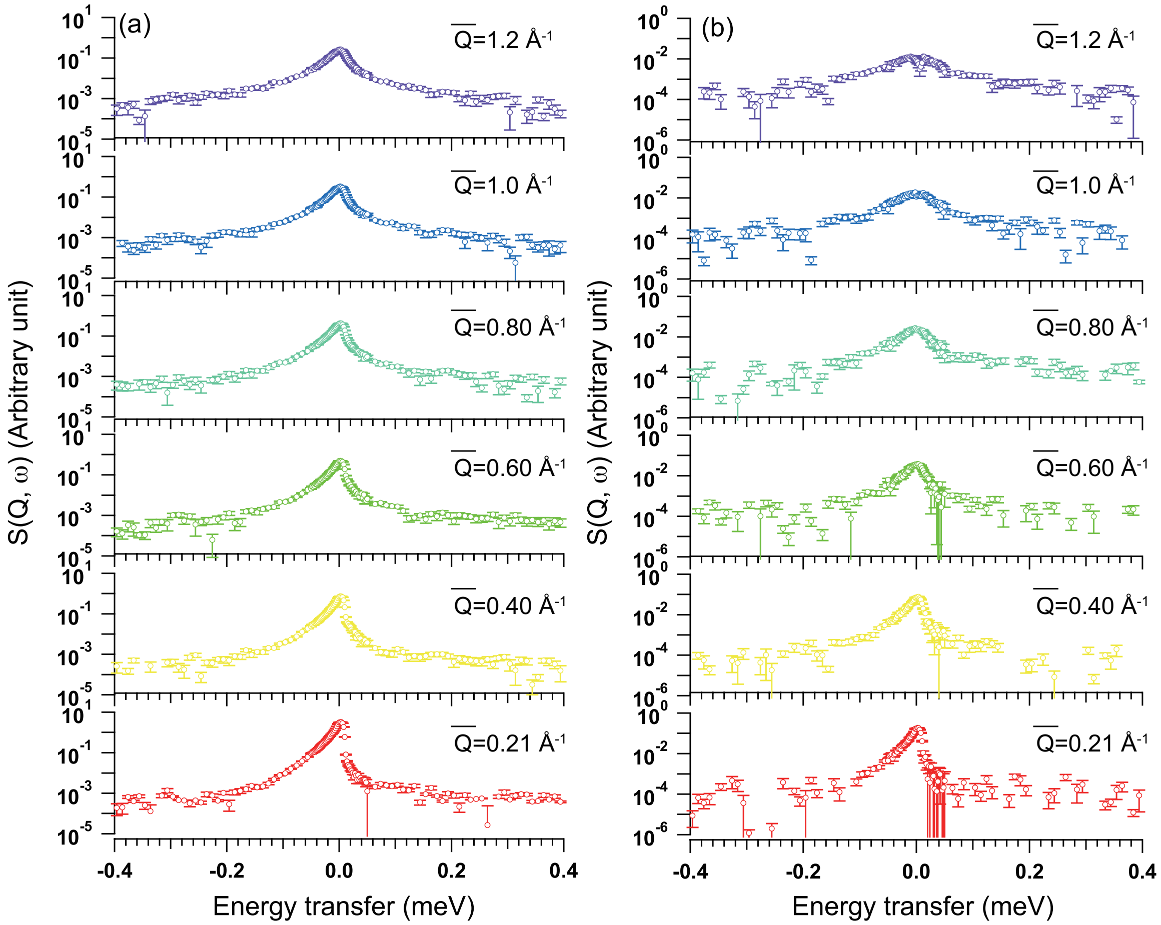

Fig. S2. $S(Q, \omega)$s from MurD and Hef-IDR.

(a) $S(Q, \omega)$ of MurD at $\overline{Q}$=0.21 Å^{-1} (red), $\overline{Q}$=0.40 Å^{-1} (yellow), $\overline{Q}$=0.60 Å^{-1} (green), $\overline{Q}$=0.80 Å^{-1} (light green), $\overline{Q}$=1.0 Å^{-1} (blue) and $\overline{Q}$=1.2 Å^{-1} (purple), respectively. (b) $S(Q, \omega)$ of Hef-IDR at $\overline{Q}$=0.21 Å^{-1} (red), $\overline{Q}$=0.40 Å^{-1} (yellow), $\overline{Q}$=0.60 Å^{-1} (green), $\overline{Q}$=0.80 Å^{-1} (light green), $\overline{Q}$=1.0 Å^{-1} (blue), and $\overline{Q}$=1.2 Å^{-1} (purple), respectively.

The calculation of H_t and H_r

By adopting the colloid diffusion theory[1], the high Q limit valued of hydodynamic function for translational diffusion (H_t) is expressed by following equation[1].

$$H_t=1-1.832\phi, \qquad \text{eq. (s1)}$$

where ϕ corresponds to the volume fraction of solute. The H_t value was computed to 0.99 for Hef-IDR solution at the concentration of 8.0 mg/mL, hence we adopted this value for further analysis.

Considering the concentration of MurD used for QENS measurements, it is expected that the H_t value must be significantly deviated from 1. In order to calculate it properly, we adopted following procedures. Firstly, D_t of MurD solution at the concentration of 52.0 mg/mL, which was exactly used for QENS study (D_{t52}), was determined through dynamic light scattering. D_{t52} was computed to (6.14± 0.02) $\times10^{-7}$ cm^2/s. Utilizing the structure solved by SAXS, D_t at infinitely dilute concentration (D_{t0}) was calculated to 6.42×10^{-7} cm^2/s with the aid of HYDROPRO. It is considered that the deviation of D_{t52} from D_{t0} is originated from non-negligible H_t. As a next step, we also determined $S(Q)$ of MurD at the concentration of 52.0 mg/mL in D_2O buffer through the SAXS measurements. From the result with fit with Percus Yveck funcion, $S(0)$ was calculated to 0.74. Referring to the paper by Ameseder et al.[1], H_t is given by a following equation.

$$H_t=(D_{t52}/D_{t0})S(0) \qquad \text{eq. (s2)}$$

Then, H_t of MurD at the concentration of 52.0 mg/mL was found to be 0.71.

Concerning about rotational diffusion (D_r), it is considered that coupling of hydrodynamics to rotational diffusion is weak[2]. Hence, we adopted the hydrodynamic function to rotational diffusion (H_r) as 1.0 for both samples.

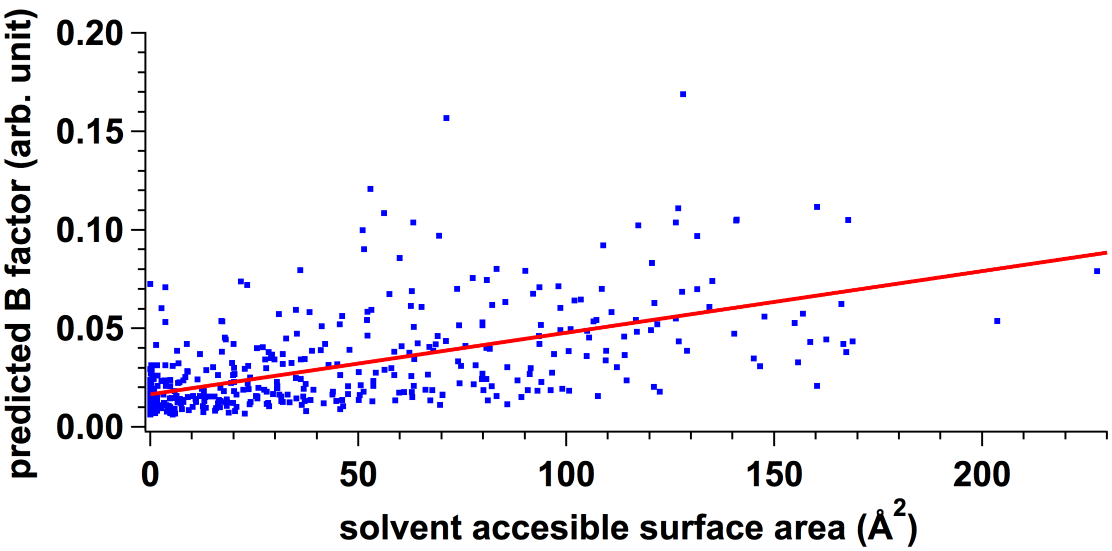

Fig. S3. The relationship between the predicted B factor and solvent accessible surface area for MurD.

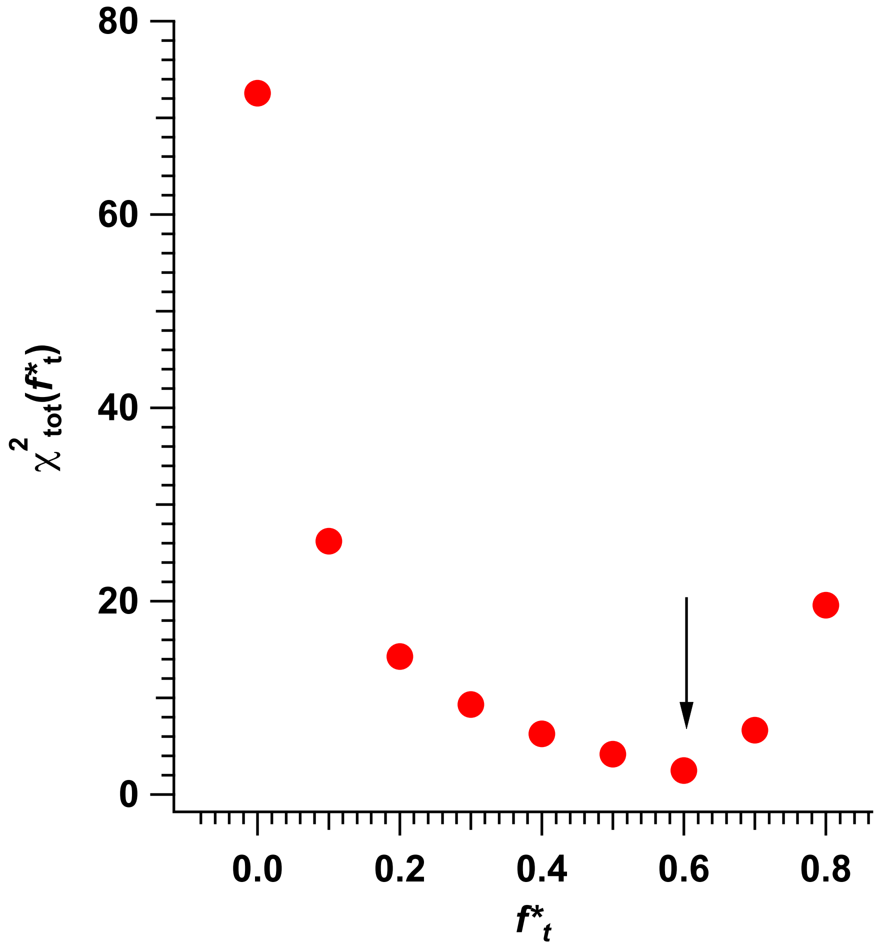

Fig. S4. χ^2_{tot} as a function of f^*_t.

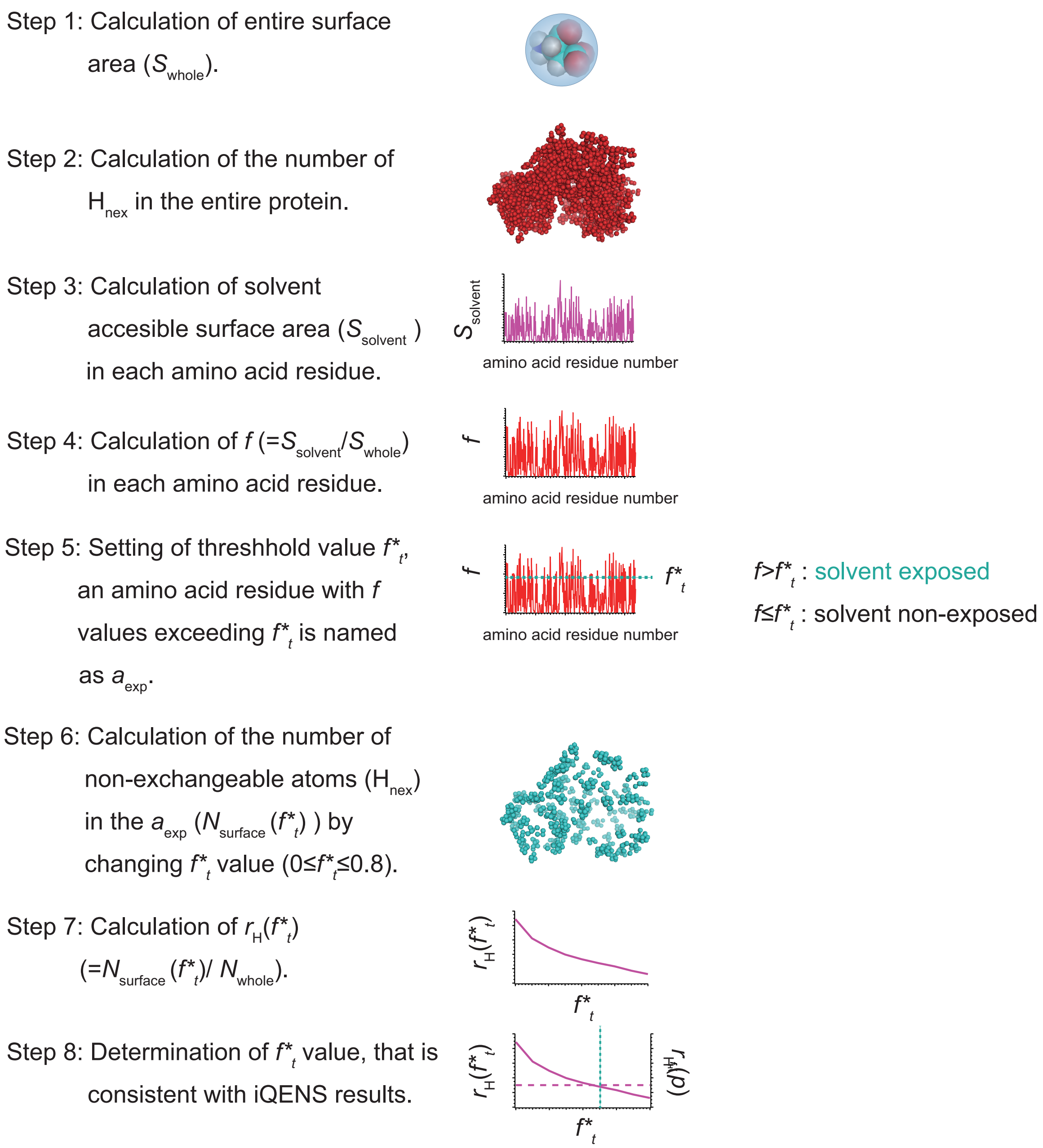

Fig. S5. Overview of determination of highly solvent exposed amino acid residues that is consistent with iQENS results.

References

1. Ameseder, F., Radulescu, A., Khaneft, M., Lohstroh, W., Stadler, A. M., Homogeneous and heterogeneous dynamics in native and denatured bovine serum albumin, *Phys. Chem. Chem. Phys*. **20**, 5128-5139 (2018).
2. Inoue, R. et al., Large Domain Fluctuations on 50-ns Timescale Enable Catalytic Activity in Phosphoglycerate Kinase, *Biophys. J*. **99**, 2309-2317 (2010).
